# Reducing Undernutrition through Counseling on Diversified Food Intake among Adult People Living with HIV on HAART, Northern Ethiopia

**DOI:** 10.1155/2020/9858619

**Published:** 2020-04-23

**Authors:** Weldegebrial Hayelom Tedla, Alemseged Aregay, Kidanu Gebremariam, Mulugeta Woldu Abrha, Haftom Gebrehiwot Weldearegay

**Affiliations:** ^1^Mekelle General Hospital, Mekelle, Ethiopia; ^2^Mekelle University, College of Health Sciences, Mekelle, Ethiopia; ^3^Tigray Health Research Institute, Mekelle, Ethiopia

## Abstract

**Background:**

HIV/AIDS and malnutrition are interrelated and exacerbate one another in a vicious cycle. As HIV infection progresses it causes catabolic state and increases susceptibility to other infections, leading to progressive aggravation of undernutrition. However, data are lacking in Ethiopia on determinants of undernutrition among people living with HIV on antiretroviral therapy. Therefore, this study aimed to assess determinants of undernutrition among adult HIV/AIDS patients in Northern Ethiopia.

**Method:**

Facility-based unmatched case-control study was conducted among 324 randomly selected people living with HIV on antiretroviral therapy (ART). A structured and pretested interviewer questionnaire was used to collect data, while digital Seca weight and Seca measuring rod were used to measure weight and height, respectively. Logistic regression was used to identify independent factors of undernutrition, and *p* value <0.05 was declared for statistical significance. All statistical analyses were performed using SPSS 21™.

**Result:**

This study revealed that people of younger age and those on ART (AOR = 0.29 (95% CI: 0.10, 0.84)) had low risk of being undernourished. However, average individual monthly income (AOR = 2.61 (95% CI: 1.48, 4.61)), not receiving nutritional counseling during visits (AOR = 2.5 (95% CI: 1.52–3.89)), and low diet diversity (AOR = 10.55 (95% CI: 4.17, 26.73)) had higher odds of undernutrition among people living with HIV/AIDS.

**Conclusion:**

Age of patients, average monthly income, nutritional counseling during visits, and diet diversity were the independent factors of undernutrition. Counseling on well-timed and sufficient consumption of nutritious foods, economic strengthening, and livelihood activities is important. Future longitudinal study is necessary to elucidate the problem of undernutrition among people living with HIV/AIDS.

## 1. Background

Globally, out of 36.9 million people living with human immunodeficiency virus (HIV), only 59% were receiving antiretroviral treatment in 2017, and around 795 million people were undernourished. Particularly, sub-Saharan Africa (SSA) remains the worst hit region with AIDS, and 23.2% of the HIV-infected was undernourished. Ethiopia had 380,000 estimated numbers of people living with HIV in Urban areas, with an adult prevalence of 3% with 98.6% of them receiving highly active antiretroviral treatment (HAART) in 2018. New infections of HIV are decreasing every year. However, people ever enrolled on ART continue increasing significantly [1–5].

The aim of the Ethiopian Growth and Transformation Plan Two, which is aligned with sustainable development goals (SDGs), is to attain economic growth of 11% per year. Although Ethiopia has witnessed improvements over the past decades, food insecurity and malnutrition are yet serious challenges [6]. Specifically prevalence of undernutrition in Ethiopia ranges from 12%–34% among HIV/AIDS patients [7, 8]. In addition, the impact of HIV on nutrition has remained a concern as they interact in a vicious cycle [9].

Malnutrition induces immunodepression and modulates the immunological response to HIV infection, affecting the overall clinical outcome and worsening HIV-related immune depression. In turn, people living with HIV/AIDS on HAART have increased requirements for macro- and micronutrients, increased metabolic demand, and low appetite which may lead to decreased immunity which in turn may lead to further undernutrition [10–14]. Simultaneously, undernutrition exacerbates drug side effects [15–18] and alters drug mechanism [19–21].

Despite there being a growing number of HAART users (98.6% of HIV patients are on HAART) and significant efforts to minimize incidence of HIV in Ethiopia, nutritional status of patients living with HIV gets insufficient attention and there is limited evidence about factors affecting nutritional condition of people living with HIV/AIDS on HAART.

Therefore, this study assessed determinants of undernutrition among adult HIV/AIDS patients on HAART at Mekelle city, Northern Ethiopia. The results will help program managers and decision makers to design nutritional intervention in accordance with local resources, programmatic, and clinical experience and can support future studies.

## 2. Methods

### 2.1. Study Setting and Population

This facility-based unmatched case-control study was conducted from March to May, 2017, in HAART centers (Mekelle Hospital, Ayder Referral Hospital, Quiha Hospital, Mekelle Health Center, Kasech Health Center, and Semen Health Center) in Mekelle city which is the capital city of Tigray region, Ethiopia. All PLWHA on HAART followed up for greater than three months in public health facilities were the source population, and people with the age of 18 years and above currently receiving ART were included from all ART clinics.

### 2.2. Sample Size and Sampling Technique

Sample size was calculated using Open Epi-3.1 with the following assumption: 95% CI, 80% power, percent of exposure among cases (39%) and controls (24.5%) [22], a case to control ratio of 1 : 1, and 10% nonresponse. This gave a total sample size of 324. The number of study participants was allocated for each HAART center based on population proportion to size allocation. Finally, cases and controls were selected based on nutritional status of the patient measured using BMI, of which patients whose BMI less than 18.5 kg/m^2^ were assigned as a potential case, whereas patients whose BMI greater than 18.5 kg/m^2^ were categorized as a potential control. Cases were selected randomly using a computer-generated simple random number based on patient HAART unique identification number. Random selection of cases was done prior to patients presenting to clinic. Then, for each successfully enrolled case, control that arrived to ART follow-up at the same clinic was enrolled as a control.

### 2.3. Measurement and Variable

The outcome variable was undernutrition measured through body mass index (BMI). To calculate BMI, height was measured using Seca measuring rod calibrated to 0.5 cm. Weights were measured using standardized digital Seca weight scale calibrated to 0.1 kg. The weight and height were converted to BMI using SPSS version 21 by dividing weight by height squared. The body weight was taken while subject wore light clothes and shoes taken off. Similarly, height measurements was carried out while the subject removed his/her shoes, stood erect, looking straight in a horizontal plane with feet together and knees straight.

Explanatory characteristics were socioeconomic and demographic variables, nutritional-related factors, and environmental-related factor.

Tools for measuring food security status were adopted from the Household Food Insecurity Access Scale guideline developed by USAID [23]. The questionnaire usually employs a series of nine questions that detect the level of concern and the lack of access to variety and/or quantity of food. The four weeks of recall period was used. If the answer is “yes” to an occurrence question, a frequency of occurrence question was asked to determine whether the condition happened rarely (once or twice), sometimes (three to ten times), or often (more than ten times) [24].

Determination of individual Dietary Diversity Score (IDDS) was done by asking the respondents to list all the food items consumed in the previous 24 hour preceding the survey date. Then, the reported food items were classified based on ten [25] food groups to identify whether they consume adequate, moderate, or inadequate micronutrient to know its adequacy level [26].

The anemia status of the study participants was ascertained using the standard criteria for female and male adults. Accordingly, the hemoglobin level for female and male greater than 12.0 gm/dl and 13.0 gm/dl, respectively, is considered nonanemic, 11–11.9 mg/dl for female and 11–12.9 mg/dl for male is considered as mild anemia, 8–10.9 mg/dl for both female and male is considered as moderate anemia, while lower than 8 mg/dl is considered as severely anemic [27].

### 2.4. Data Collection Procedure and Quality Control Mechanism

Research assistances were trained for two days on interviewing technique and questionnaire content. Research questionnaire and data extraction format was prepared from EDHS and other literature studies. For consistency, the questionnaire was prepared in English and translated to Tigrigna then retranslated to English. Data were collected using pretested questionnaire by face-to-face interview, and laboratory results and relevant medical data were retrieved from medical files. Daily supervision, spot checking, and reviewing completed questionnaire were conducted.

### 2.5. Data Analysis

Data were entered in to EPI-info version 7, cleaned, and analyzed using SPSS version 21. Cross tabulation (frequency with percentage) was used to show results of categorical variables. Chi-square was used to see the association between dependent variable and each independent variable. Finally, multivariable logistic regression was used to see the predictors of the outcome variable. Variables with *p* value <0.25 at bivariable analysis were further analyzed in the multivariable logistic regression. Statistical significance for the association was declared at *p* value <0.05. Multicolinearity was checked by standard error. The model adequacy was checked by using Hosmer and Lemeshow goodness of fit test.

## 3. Results

### 3.1. Sociodemographic Characteristics of the Study Participants

Table 1 shows the sociodemographic characteristics of the 324 HIV-positive adults on ART enrolled in this study. Above two-thirds of 112 (69.1%) the cases and 103 (63.6%) controls were females. Regarding the age of the patients, 6 out of ten (*n* = 62) of the cases and 4 out of ten of the controls were in the age range of 30–39 years. Thirty-eight percent (*n* = 63) of the cases were divorced, and 46.9 percent (*n* = 76) of the controls were married. Concerning the educational status, two-fifth 63 (38.9%) of the cases and quarter (*n* = 41) of the controls were illiterate. A majority of the cases and controls were Orthodox and daily laborer by occupation. A majority of the cases 100 (64.5%) had mean monthly income of above 1200 ETB (57 USD), and 107 (63.3%) of the controls had below 1200 ETB (57 USD).

### 3.2. Nutrition-Related Characteristics of the Study Participants

Eighty-four percent (*n* = 136) of the cases and eight of ten (*n* = 130) controls had received nutritional counseling. Regarding the frequency of counseling majority of the cases and controls, 94 (69.1%) and 78 (60%) had got counseling during every visit to the facility, respectively. Slightly above half of the 87 (53.7%) cases and two-third (103) (63.6%) of the controls did not have food support. Data on the household food insecurity and dietary diversity status showed that 95% (*n* = 155) of cases and 80% (*n* = 130) of controls were food insecure, and 47% (*n* = 77) of the cases and 63.36% (*n* = 103) of the controls had medium diet diversity score Table 2.

### 3.3. Environment-Related Factors of the Study Participants

Participants drinking water from protected source in cases and controls were 141 (87%) and 150 (92.6%), respectively. Eigth out of ten of 134 (82.7%) cases and 145 (89.5%) controls were using protected water for food preparation. A majority of the cases 145 (89.5%) and 157 (96.9%) had access to a latrine, and forty-three percent (*n* = 63) of the cases had pit latrine and 69 (43.9%) of the controls had flash latrine. Six out of ten of cases (*n* = 87) and above half or 84 (53.5%) did not have a hand washing facility in the latrine Table 3.

### 3.4. Factors Associated with Undernutrition

Out of 16 selected variables that showed association with undernutrition in the bivariable model, only four variables (age in completed years, household income, nutritional counseling, and diet diversity) were significantly associated with under nutrition during multivariable logistic regression analysis Table 4.

Compared to greater than 50 years old on ART, those of younger age had less odds of being undernourished; 18–29 years (AOR = 0.29 (95% CI: 0.10, 0.84)), 30–39 years (AOR = 0.31 (95% CI: 0.13, 0.76)) and 40–49 years (AOR = 0.35 (95% CI: 0.14, 0.86)). The odds of having below average monthly income (mean = 1200 ETB) of people living with HIV on HAART were 2.6 times higher among cases than the controls (AOR = 2.61 (95% CI: 1.48, 4.61)). Likewise, those who did not got nutritional counseling during visits were 2.5 times more undernourished than their counterparts (AOR = 2.5 (95% CI: 1.52–3.89)). Low diet diversity score was associated with a tenfold higher odds of being undernourished when compared with those with higher IDDS (AOR = 10.55 (95% CI: 4.17, 26.73)). No significant differences were noted in risk factors for under nutrition when stratified by water source and having access of latrine Table 4.

Two additional regression analyses were performed looking at the dependent variable in order to evaluate the potential confounding effect of inclusion proximal and distal causes of undernutrition in the same regression model. Income, food support, family size, and educational status were independent factors affecting food insecurity. And also, diet diversity was significantly affected by economic status, education level, residence, and nutritional counseling Tables 5 and 6.

## 4. Discussion

HIV infection has long been associated with wasting syndrome and underweight. This study found that age of the patients in completed year, average individual monthly income, nutritional counseling, and diet diversity were the strongest factors of undernutrition among people with HIV on ART.

This study uncovered that younger age had less risk of being undernourished when compared with older age patients. This result is consistent with study done in Tanzania [28], Zimbabwe [29] which indicates that younger people living with HIV on ART might have higher immunity and can work by themselves to get foods in their everyday lives and also starting ART at younger age could diminish the impact of the virus early. But this result is unsupported by a study done in Ethiopia [30] in which the older aged HIV-positive clients were at lower risk of developing undernutrition. This could be due to differences in sampling and recruiting of the study participants.

Other finding was people living with HIV on ART having below average monthly income (mean = 1200 ETB) had higher odds of cases. This is in line with a study done in Kenya [31], Tigray Region, Ethiopia [32], Democratic Republic of Congo [33], Uganda [34], Dilla University Referral Hospital, Ethiopia [7], and Zimbabwe [29] which indicates that poor socioeconomic factors leads to lack of access to food and is associated with undernutrition among people living with HIV enrolled on antiretroviral treatment. This has significant programmatic implication; in that addressing the poverty, food insecurity issues, and ensuring financial stability among PLWHA is a significant aspect in achieving better clinical outcome.

Another finding showed that HIV patients with inadequate diet diversity had higher odds of being undernutritious. This result is congruent with a study done in Ethiopia [35, 36] and Kenya [37] which revealed that lack of food diversity is an important issue, particularly in developing countries where diets consist of mainly starchy staples with less access to nutrient-rich food [38].This is because dietary diversity has been recognized as an indicator of food security, with consumption of more food groups suggesting better nourishment and improved micronutrient intake. But this study was incoherent with study done in Rwanda [39], this contradiction could be due to the difference in assessment tool in which the study in Rwanda used inappropriate single question to assess food insufficiency. Nutrition interventions that educate low-income families on inexpensive, healthy eating should be embraced, and changes at policy level should be well planned to raise affordability and accessibility of healthful food in Ethiopia.

Also, this study indicated that people living with HIV on ART who did not receive nutritional counseling were associated with undernutrition. This is similar to studies from Ethiopia [40], India [41], and Honduras [42], in which nutritional counseling improves dietary feeding practices. This implies that culturally suitable and sustainable interventions that provide nutrition counseling for people on ART and of diverse nutritional statuses are warranted.

We conducted separate analyses to evaluate the consistency of our findings by looking at factors associated with two potential mediators of undernutrition: diet diversity and food security. The findings from these additional analyses were consistent with our primary analysis, but also highlighted the importance of education, income, and dietary counseling on diet diversity, as well as education, income, family size, and food support on food security, two important proximal causes of undernutrition.

The strength of this study includes adequate sample. The weight of study participants was measured using standardized and calibrated weight scales even as height was measured using standardized height boards. A standardized questionnaire was also administered by trained data collectors. The shortcoming of this study was recall bias by study respondents on the type of foods consumed in their households. This may have led to incorrect calculation and misclassification of the household food consumption score.

## 5. Conclusion

In our finding, younger age, low individual monthly income, not receiving nutritional counseling, and having low diet diversity were the determinants of undernutrition. Providing counseling on well-timed and adequate consumption of nutritious foods and strengthening income generating activities are important to ensure that PLWHIV are able to address the underlying causes of undernutrition. Escalating nutrition assessment, counseling and support (NACS) during visit to the facilities, and increasing effective cross-sectoral integrated programs will improve nutritional status. Furthermore, longitudinal research is needed to explore the problem of undernutrition among HIV/AIDS.

## Figures and Tables

**Table 1:** Sociodemographic characteristics of adult HIV patient attending treatment, 2017 (*n* = 324).

Characteristics	Undernutrition, *n* (%)		Total
	Case	Control	
Sex of respondent			
Male	50 (30.8)	59 (36.4)	109 (33.6)
Female	112 (69.1)	103 (63.6%)	215 (66.3)

Age in completed years			
18–29	21 (13)	28 (17.3)	49 (15.1)
30–39	62 (38.3)	70 (43.2)	132 (40.7)
40–49	50 (30.9)	47 (29)	97 (29.9)
≥50	29 (17.9)	17 (10.5)	46 (14.2)

Residence			
Urban	127 (78.4)	139 (85.8)	266 (82.1)
Rural	35 (21.6)	23 (14.2)	58 (17.9)

Marital status			
Married	57 (35.2)	76 (46.9)	133 (41)
Unmarried	19 (11.7)	10 (6.2)	29 (9)
Divorced	63 (38.9)	60 (37)	123 (38)
Widowed	23 (14.2)	16 (9.9)	39 (12)

Educational status			
Illiterate	63 (38.9)	41 (25.3)	104 (32.1)
Read and write	28 (17.3)	38 (23.5)	66 (20.4)
Primary education	28 (17.3)	42 (25.9)	70 (21.6)
Secondary education	29 (17.9)	29 (17.9)	58 (17)
Above grade 12	14 (8.)	12 (7.4)	26 (8)

Religion			
Orthodox	156 (96.3)	147 (90.7)	303 (93.5)
Muslim	6 (3.7)	15 (9.3)	21 (6.5)

Occupation			
Farmer	31 (19.1	20 (12.3)	51 (15.7)
Employed	28 (17.3)	27 (16.7)	55 (17)
Merchant	26 (16)	35 (21.6)	61 (18.8)
Daily laborer	51 (31.5)	46 (28.4)	97 (18.5)
Unemployed	26 (16)	34 (21.0)	60 (18.5)

Individual monthly income (mean)			
>1200 ETB	100 (64.5)	55 (36.7)	155 (47.5)
<1200 ETB	62 (36.7)	107 (63.3)	169 (52.2)

Family size			
≤ 5 members	129 (79.6)	132 (81.4)	261 (80.6)
>5 members	33 (20.3)	30 (18.5)	63 (19.4)

**Table 2:** Nutrition-related characteristics of adult HIV patient attending treatment 2017, (*N* = 324).

Characteristics	Undernutrition, *n* (%)		Total
	Case	Control	
Dietary/nutrition counseling			
Yes	136 (84)	130 (80.2)	266 (82.1)
No	26 (16)	32 (19.8)	58 (17.9)

Frequency of counseling			
Every visit	94 (69.1)	78 (60)	172 (64.7)
Sometime	33 (24.3)	46 (35.4)	79 (29.7)
Rarely	9 (6.6)	6 (4.6)	15 (5.6)

Food support			
Yes	75 (46.3)	59 (36.4)	134 (41.4)
No	87 (53.7)	103 (63.6)	190 (58.6)

Food support from			
Family	1 (1.7)	6 (10.2)	7 (5.2)
Government	35 (46.7)	15 (25.4)	50 (37.3)
NGO	39 (52)	38 (64.4)	77 (57.5)

Food security			
Secured	7 (4.3)	32 (19.8)	39 (12)
Insecure	155 (95.7)	130 (80.2)	285 (88)

Diet diversity score			
Low	72 (44.4)	14 (8.6)	238 (73.5)
Medium	77 (47.5)	103 (63.6)	86 (26.5)
High	13 (8.0)	45 (27.8)	

**Table 3:** Environment-related factor of adult HIV patient in Mekelle ART center attending treatment, 2017.

Characteristics	Undernutrition, *n* (%)		Total
	Case	Control	
Source of water for drinking			
Protected	141 (87)	150 (92.6)	291 (89.8)
Unprotected	21 (13)	12 (7.4)	33 (10.2)

Water supply for food preparation			
Protected	140 (88.1)	151 (94.4)	291 (91.2)
Unprotected	19 (11.9)	9 (5.6)	28 (8.8)

Store of water (common)			
Plastic container	134 (82.7)	145 (89.5)	279 (86.1)
Pot	1 (0.6)	2 (1.2)	3 (0.9)
Bucket	22 (13.6)	8 (4.9)	30 (9.3)
Barrel	5 (3.1	7 (4.3)	12 (3.7)

Accesses latrine			
Yes	145 (89.5)	157 (96.9)	302 (93.2)
No	17 (10.5)	5 (3.1)	22 (6.8)

Type of latrine			
Pit	63 (43.4)	57 (36.3)	120 (39.7)
Flash latrine	50 (34.5)	69 (43.9)	119 (39.4)
VIP	32 (22.1)	31 (19.7)	63 (20.9)

Hand washing facility at toilet			
Yes	58 (40)	73 (46.5)	131 (43.4)
No	87 (60)	84 (53.5)	171 (56.6)

**Table 4:** Factors associated with under nutrition among adult HIV patients attending treatment, 2017 (*N* = 324).

Characteristics	Undernutrition, *n* (%)		Pearson Chi-square	OR (95% CI)
	Case	Control	*X* ^2^ (*p* value)	Adjusted
Age in completed years				
18–29	21 (13)	28 (17.3)	4.70 (0.19)	0.29 (0.10–0.84)
30–39	62 (38.3)	70 (43.2)		0.31 (0.13–0.76)
40–49	50 (30.9)	47 (29)		0.35 (0.14–0.86)
≥50	29 (17.9)	17 (10.5)		1

Residence				
Urban	127 (78.4)	139 (85.8)	3.02 (0.08)	NS
Rural	35 (21.6)	23 (14.2)		

Marital status				
Married	57 (35.2)	76 (46.9)	6.84 (0.08)	NS
Unmarried	19 (11.7)	10 (6.2)		
Divorced	63 (38.9)	60 (37)		
Widowed	23 (14.2)	16 (9.9)		

Educational status				
Illiterate	61 (37.7)	42 (25.9)	6.19 (0.05)	NS
Read and write	28 (17.3)	41 (25.3)		
Formal education	73 (45.1)	79 (48.8)		

Occupation				
Farmer	31 (19.1	20 (12.3)	5.04 (0.28)	NS
Employed	28 (17.3)	27 (16.7)		
Merchant	26 (16)	35 (21.6)		
Daily laborer	51 (31.5)	46 (28.4)		
No job	26 (16)	34 (21.0)		

Income (ETB), mean (1200)				
Below mean	100 (64.5)	55 (3535)	25.05 (0.00)	2.61 (1.48–4.61)
Above mean	62 (36.7)	107 (63.3)		1

Family size				
≤5 members	129 (79.6)	132 (81.4)	0.05 (0.82)	NS
>5 members	33 (20.3)	30 (18.5)		

Dietary/nutrition counseling				
Yes	136 (84)	130 (80.2)	0.76 (0.38)	1
No	26 (16)	32 (19.8)		2.50 (1.52–3.89)

Frequency of counseling				
Every visit	94 (69.1)	78 (60)	4.25 (0.12)	NS
Some times	33 (24.3)	46 (35.4)		
Rarely	9 (6.6)	6 (4.6)		

Food support				
Yes	75 (46.3)	59 (36.4)	3.26 (0.07)	NS
No	87 (53.7)	103 (63.6)		

Food security				
Secured	7 (4.3)	32 (19.8)	3.03 (0.08)	NS
Insecure	155 (95.7)	130 (80.2)		

Diet diversity score				
Low	72 (44.4)	14 (8.6)	60.53 (0.00)	10.55 (4.17–26.73)
Medium	77 (47.5)	103 (63.6)		1.90 (0.87–4.11)
High	13 (8.0)	45 (27.8)		1

Source of water for drinking				
Protected	141 (87)	150 (92.6)	2.73 (0.09)	NS
Unprotected	21 (13)	12 (7.4)		

Water supply for food preparation				
Protected	140 (88.1)	151 (94.4)	4.08 (0.04)	NS
Unprotected	22 (13.6)	11 (6.8)		

Store of water (common)				
Plastic container/pot	135 (83.3)	147 (90.7)	3.93 (0.05)	NS
Bucket/barrel	27 (16.7)	15 (9.3)		

Accesses latrine				
Yes	145 (89.5)	157 (96.9)	7.02 (0.01)	NS
No	17 (10.5)	5 (3.1)		

Type of latrine				
Pit	63 (43.4)	57 (36.3)	2.88 (0.24)	NS
Flash latrine	50 (34.5)	69 (43.9)		
VIP	32 (22.1)	31 (19.7)		

Hand washing facility at toilet				
Yes	58 (40)	73 (46.5)	1.30 (0.26)	NS
No	87 (60)	84 (53.5)		

NS represents nonsignificant, bivariate analysis is done using Pearson Chi-square.

**Table 5:** Factors associated with diet diversity among adult HIV patients attending treatment, 2017 (*N* = 324).

Characteristics	Diet diversity, *n* (%)		Pearson Chi-square	OR (95% CI)
	High	Medium/low	*X* ^2^ (*p* value)	Adjusted
Age in completed years				
18–29	10 (17.2)	39 (14.7)	1.37 (0.71)	NS
30–39	26 (44.8)	106 (39.8)		
40–49	16 (27.6)	81 (30.5)		
≥50	6 (10.3)	40 (15)		

Residence				
Urban	51 (87.9)	215 (80.8)	1.63 (0.20)	1.50 (1.34–1.69)
Rural	7 (12.1)	51 (19.2)		1

Marital status				
Married	30 (57.7)	103 (38.7)	3.55 (0.32)	NS
Unmarried	5 (8.6)	24 (9)		
Divorced	18 (31)	105 (39.5)		
Widowed	5 (8.6)	34 (12.8)		

Educational status				
Illiterate	15 (25.9)	88 (33.1)	1.17 (0.55)	1
Read and write	13 (22.4)	56 (21.1)		1.03 (1.01–1.09)
Formal education	30 (51.7)	122 (45.9)		1.23 (1.02–1.34)

Occupation				
Farmer	7 (12.1)	44 (16.5)	6.05 (0.19)	NS
Employed	16 (27.6)	39 (14.7)		
Merchant	9 (15.5)	52 (19.5)		
Daily laborer	17 (29.9)	80 (30.1)		
No job	9 (15.5)	51 (19.2)		

Income (ETB), mean (1200) (57 USD)				
Below mean	23 (30.3)	146 (54.9)	4.42 (0.03)	1
Above mean	35 (60.3)	120 (45.1)		3.05 (2.79–3.12)

Family size				
≤5 members	55 (94.8)	248 (93.2)	0.20 (0.65)	NS
> 5 members	3 (3.8)	18 (6.8)		

Dietary/nutrition counseling				
Yes	49 (84.5)	217 (81.6)	0.27 (0.60)	1.52 (1.34–1.70)
No	9 (15.5)	49 (18.4)		1

Food support				
Yes	22 (37.9)	112 (42.1)	0.34 (0.55)	NS
No	36 (62.1)	154 (57.9)		

Source of water for drinking				
Protected	55 (94.8)	236 (88.7)	1.9 (0.16)	NS
Unprotected	3 (5.2)	30 (11.3)		

Water supply for food preparation				
Protected	55 (94.8)	236 (88.7)	1.9 (0.16)	NS
Unprotected	3 (5.2)	30 (11.3)		

Store of water (common)				
Plastic container/pot	54 (93.1)	228 (85.7)	2.3 (0.13)	NS
Bucket/barrel	4 (6.9)	38 (14.3)		

Accesses latrine				
Yes	56 (93.2)	246 (92.5)	1.24 (0.26)	NS
No	2 (3.4)	20 (7.5)		

Type of latrine				
Pit	22 (39.3)	98 (39.8)	0.01 (0.99)	NS
Flash latrine	22 (39.3)	97 (39.4)		
VIP	12 (21.4)	51 (20.7)		

Hand washing facility at toilet				
Yes	28 (50)	103 (41.9)	1.22 (0.27)	NS
No	28 (50)	143 (58.1)		

NS represents nonsignificant, bivariate analysis is done using Pearson Chi-square.

**Table 6:** Factors associated with food insecurity among adult HIV patients attending treatment, 2017 (*N* = 324).

Characteristics	Food security, *n* (%)		Pearson Chi-square	OR (95% CI)
	Secured	Insecure	*X* ^2^ (*p* value)	Adjusted
Age in completed years				
18–29	46 (16.1)	3 (7.7)	4.15 (0.24)	NS
30–39	117 (41.1)	15 (38.5)		
40–49	85 (29.8)	12 (30.8)		
≥50	37 (13)	9 (23.1)		

Residence				
Urban	234 (82.1)	32 (82.1)	0.00 (0.99)	NS
Rural	51 (17.9)	7 (17.9)		

Marital status				
Married	120 (42.1)	13 (33.3)	3.42 (0.33)	NS
Unmarried	25 (8.8)	4 (10.3)		
Divorced	109 (38.2)	14 (35.9)		
Widowed	31 (34.3)	8 (20.5)		

Educational status				
Illiterate	93 (32.6)	10 (25.6)	1.61 (0.04)	1.71 (1.52–1.85)
Read and write	62 (21.8)	7 (17.9)		1.10 (0.99–1.35)
Formal education	130 (45.6)	22 (56.4)		1

Occupation				
Farmer	48 (16.8)	3 (7.7)	4.07 (0.39)	NS
Employed	47 (16.5)	8 (20.5)		
Merchant	53 (18.6)	8 (20.5)		
Daily laborer	82 (28.8)	15 (38.5)		
No job	55 (19.3)	5 (12.8)		

Income (ETB), mean (1200)				
Below mean	145 (50.9)	24 (61.5)	1.56 (0.21)	1.89 (1.71–2.02)
Above mean	140 (49.1)	15 (38.5)		1

Family size				
≤5 members	265 (92.9)	28 (71.8)	0.52 (0.05)	0.40 (0.39–0.41)
>5 members	20 (7.1)	11 (28.2)		1

Dietary/nutrition counseling				
Yes	233 ()	33 (84.6)	0.19 (0.66)	NS
No	52 (18.2)	6 (15.4)		

Food support				
Yes	119 (41.8)	15 (38.5)	0.15 (0.69)	0.25 (0.20–0.31)
No	166 (58.2)	24 (61.5)		1

Source of water for drinking				
Protected	254 (89.1)	37 (94.9)	1.23 (0.26)	NS
Unprotected	31 (10.9)	2 (5.1)		

Water supply for food preparation				
Protected	254 (89.1)	37 (94.9)	1.23 (0.26)	NS
Unprotected	31 (10.9)	2 (5.1)		

Store of water (common)				
Plastic container/pot	249 (87.4)	33 (84.6)	0.23 (0.63)	NS
Bucket/barrel	36 (12.6)	6 (15.4)		

Accesses latrine				
Yes	266 (93.3)	36 (92.3)	0.05 (0.81)	NS
No	19 (6.7)	3 (7.7)		

Type of latrine				
Pit	108 (40.6)	12 (33.3)	1.09 (0.57)	NS
Flash latrine	102 (38.3)	17 (47.2)		
VIP	56 (21.1)	7 (19.4)		

Hand washing facility at toilet				
Yes	106 (40.3)	25 (64)	0.37 (0.26)	NS
No	157 (59.7)	14 (36)		

NS represents nonsignificant, bivariate analysis is done using Pearson Chi-square.

## Data Availability

The datasets used and/or analyzed during the current study are available from the corresponding author on request.

## Abbreviations

ART: Antiretroviral therapy
BMI: Body mass index
IDDS: Individual diet diversity score
EDHS: Ethiopian Demographic Health Survey
PLWHA: People living with HIV/AIDS.
